# Characterization of Two Novel Bacteriophages Infecting Multidrug-Resistant (MDR) *Acinetobacter baumannii* and Evaluation of Their Therapeutic Efficacy *in Vivo*

**DOI:** 10.3389/fmicb.2018.00696

**Published:** 2018-04-10

**Authors:** Kyoungeun Cha, Hynu K. Oh, Jae Y. Jang, Yunyeol Jo, Won K. Kim, Geon U. Ha, Kwan S. Ko, Heejoon Myung

**Affiliations:** ^1^Department of Bioscience and Biotechnology, Hankuk University of Foreign Studies, Yong-In, South Korea; ^2^The Bacteriophage Bank of Korea, Hankuk University of Foreign Studies, Yong-In, South Korea; ^3^Samsung Medical Center, Sungkyukwan University School of Medicine, Suwon, South Korea

**Keywords:** multidrug-resistance, *Acinetobacter baumannii*, bacteriophage therapy, mouse model, genome analysis

## Abstract

*Acinetobacter baumannii* is emerging as a challenging nosocomial pathogen due to its rapid evolution of antibiotic resistance. We report characterization of two novel bacteriophages, PBAB08 and PBAB25, infecting clinically isolated, multidrug-resistant (MDR) *A. baumannii* strains. Both phages belonged to Myoviridae of Caudovirales as their morphology observed under an electron microscope. Their genomes were double stranded linear DNAs of 42,312 base pairs and 40,260 base pairs, respectively. The two phages were distinct from known *Acinetobacter* phages when whole genome sequences were compared. PBAB08 showed a 99% similarity with 57% sequence coverage to phage AB1 and PBAB25 showed a 97% similarity with 78% sequence coverage to phage IME_AB3. BLASTN significant alignment coverage of all other known phages were <30%. Seventy six and seventy genes encoding putative phage proteins were found in the genomes of PBAB08 and PBAB25, respectively. Their genomic organizations and sequence similarities were consistent with the modular theory of phage evolution. Therapeutic efficacy of a phage cocktail containing the two and other phages were evaluated in a mice model with nasal infection of MDR *A. baumannii*. Mice treated with the phage cocktail showed a 2.3-fold higher survival rate than those untreated in 7 days post infection. In addition, 1/100 reduction of the number of *A. baumannii* in the lung of the mice treated with the phage cocktail was observed. Also, inflammatory responses of mice which were injected with the phage cocktail by intraperitoneal, intranasal, or oral route was investigated. Increase in serum cytokine was minimal regardless of the injection route. A 20% increase in IgE production was seen in intraperitoneal injection route, but not in other routes. Thus, the cocktail containing the two newly isolated phages could serve as a potential candidate for therapeutic interventions to treat *A. baummannii* infections.

## Introduction

*Acinetobacter baumannii* is an opportunistic pathogen causing nosocomial infection in hospitals. It is designated as an ESKAPE (*Enterococcus faecium, Staphylococcus aureus, Klebsiella pneumonia, A. baumannii, Pseudomonas aeruginosa*, and *Enterobacter species*) pathogen by World Health Organization (WHO) (McConnell et al., 2013). It causes mainly pneumonia and additionally burn infections, meningitis, urinary tract infections, and sepsis (Dijkshoorn et al., 2007). Antibiotic- resistant strains are rapidly emerging and even multidrug-resistant (MDR) and pandrug-resistant (PDR) strains are observed (Tuon et al., 2015). Its virulence factors include porins, lipopolysaccharides, capsular polysaccharides, metal acquisition systems, phospholipases, outer membrane vesicles, and protein secretion systems (Weber et al., 2016; Lee et al., 2017). Factors influencing antibiotic resistance includes two-component systems AdeRS, BaeSR, GacSA, and PmrAB (Kröger et al., 2017). Treatment of carbapenem-resistant *A. baumannii* (CRAB) involves the use of combinations of last resort agents including colistin and tigecycline, but the efficacy and safety issues are not cleared yet (Doi et al., 2015).

Phage therapy is a promising tool for controlling drug-resistant bacteria (Abedon, 2017; Lin et al., 2017). The mechanism of drug-resistance is totally unrelated to that of phage infection. Although bacteriophages have been used as an antibacterial for almost 100 years, mainly in eastern European countries, they are not recognized as drugs due to the lack of a proper documentation needed for drug approval. In addition, we still wait for fulfillment of regulatory affairs to approve phage drugs (Huys et al., 2013).

Novel therapies including bacteriophages against drug-resistant *A. baumannii* have been reported and reviewed (Mihu and Martinez, 2011; García-Quintanilla et al., 2013; Parasion et al., 2014). Successful phage control of various strains of *Acinetobacter* was demonstrated not only *in vitro* but also *in vivo*. Two newly isolated phages infecting *A. baumannii* were characterized and suggested as potential candidates for phage cocktail (Merabishvili et al., 2014). Other two newly isolated phages were characterized at genomic DNA level and suggested as potential candidate for phage cocktail against CRAB (Jeon et al., 2016a). Phage BΦ-C62 was used to successfully control CRAB infection via nasal route in mice model (Jeon et al., 2016b). Phage *vB-GEC_Ab-M-G7* was used to successfully control wound infection in a rat model (Kusradze et al., 2016). Medes et al. tested phage cocktails against diabetic cutaneous wounds in two animal models. Phage cocktails for *Staphylococcus aureus* or *Pseudomonas aeruginosa* improved wound healing, but cocktail for *A. baumannii* was not as effective. A personalized phage cocktail for treating mouse dorsal wound model was reported (Regeimbal et al., 2016). Phages from multi-institute libraries were used to make a personalized cocktail and proven effective for treating a diabetic human patient with necrotizing pancreatitis complicated by an MDR *A. baumannii* infection (Schooley et al., 2017). A phage was effective in resolving wound infection caused by multidrug-resistant *A. baumannii* in an uncontrolled diabetic rat model (Shivaswamy et al., 2015). A bacteriophage-containing aerosol was proven effective for cleaning and decreased the rates of infection caused by CRAB in intensive care units (Wang et al., 2016). A combined lysis spectrum of four lytic phages against clinically isolated CRAB was reported to be 87.5% and phages were proven effective as therapeutic agents for lung infection without deleterious side effects in mice model (Hua et al., 2018).

Here, we report both microbiological characterization of novel phages infecting *A. baumannii* and the efficacy of a phage cocktail containing the phages in control of nasal infection in a mice model. Further, we provide the basis why the phages can be used as a therapeutic intervention practically.

## Materials and methods

### Ethics statement

All animal studies were approved by and followed the guidelines and regulations of the Ethical Committee for Animal Experiments of Hankuk University of Foreign Studies (approval number 2017-0001).

### Bacterial strains and phages used

Fourteen clinical *A. baumannii* strains were obtained from patients in Samsung Medical Center, Sungkyukwan University School of Medicine. Antibiotic resistance profile of the strains is shown in Table 1. To determine the genotypes of the *A. baumannii* isolates, Oxford scheme multilocus sequence typing (MLST) was performed as described previously (Bartual et al., 2005; http://pubmlst.org/abaumannii/info/primers_Oxford.shtml). One of the strains (strain 28) was selected and further used for mice experiment. It was resistant to both kanamycin (3.5 mg/ml, Sigma, USA) and ampicillin (5 mg/ml, Sigma, USA), and this fact was used to enumerate recovered bacteria from mice lung. The 9 bacteriophages tested against the 14 strains were PBAB05, PBAB06, PBAB07, PBAB08, PBAB25, PBAB68, PBAB80, PBAB87, and PBAB93. Bacteriophages used for making the therapeutic cocktail were PBAB08, PBAB25, PBAB68, PBAB80, PBAB93 (Bacteriophage Bank of Korea).

**Table 1:** Antibiotic resistance profile of clinically isolated *Acinetobacter baumannii* strains and their phage susceptibility.

**Antibiotics/ name of isolate**	**Sequence type (ST)**	**Imipenem**	**Meropenem**	**Ampicillin/sulbactam**	**Ceftazidime**	**Cefepime**	**Gentamicin**	**Ciprofloxacin**	**Levofloxacin**	**Trimethoprim/ Sulfamethoxazole**	**Piperacillin/tazobac**	**colistin**	**Tigecycline**	PBAB number^*^ of infecting phages
Strain 21	191	>64^**^	>64	>64	>64	>64	>64	>64	64	>64	>256	1	8	none
Strain 32-a	191	>64	>64	>64	>64	>64	>64	>64	>64	>64	>256	1	8	none
Strain F-224	1240	>64	>64	>64	>64	>64	>64	>64	>32	>64	>256	1	8	5, 6, 7, 87
Strain F-1208	357	>64	>64	>64	>64	>64	>64	>64	32	>64	>256	>64	1	8, 68, 80, 93
Strain F-1510	191	>64	>64	>64	>64	>64	>64	>64	16	>64	>256	2	8	none
Strain F-1629	357	>64	>64	32	>64	64	>64	>64	32	>64	>256	>64	8	8, 68, 80, 93
Strain 26	368	16	16	32	64	64	16	4	32	32	128	64	4	8, 68, 80,
Strain 28	368	16	16	16	64	64	16	4	32	32	128	1	8	8, 25, 68, 80, 93
Strain 32-b	357	16	16	16	64	64	16	4	32	32	128	4	2	8, 68, 80, 93
Strain 54	208	16	16	32	64	64	16	4	8	32	128	1	2	none
Strain 58	208	16	16	32	64	64	16	4	8	32	128	1	2	none
Strain 81	191	16	16	32	64	64	16	4	8	32	128	4	4	5, 6, 7, 87
Strain K20-B-667	191	>64	>64	>64	>64	>64	>64	>64	>64	>64	>256	1	8	none
Strain K20-B-890	191	8	64	>64	32	64	>64	>64	>64	>64	256	1	32	none

* *PBAB numbers according to the Bacteriophage Bank of Korea (www.phagebank.or.kr)*.

** *The numbers in each box shows the maximum concentration of each antibiotic to which tested bacteria was resistant (mg/L)*.

*Darkly shaded box means “resistant”, lightly shaded box “intermediate”, and white box “susceptible”*.

### Phage isolation and purification

Bacteriophage isolation and characterization were described thoroughly (Clokie and Kropinski, 2009a,b; Clokie et al., 2018). The isolation and characterization of phages have been described previously (Kim et al., 2013). The phages were purified using a glycerol gradient centrifugation method (Sambrook and Russell, 2006). A single plaque was used to inoculate 5 ml of a mid-exponential-phase culture of the bacterium, followed by incubation at 37°C for 3 h. A phage lysate was obtained by centrifugation at 11,000 xg for 10 min and discarding the supernatant. Five ml of the lysate was used to inoculate 100 ml of a mid-exponential-phase culture of the bacterium, and the mixture was then incubated until lysis was completed. NaCl was added to the lysate at a final concentration of 1 M, and it was incubated at 4°C for 1 h. After centrifugation at 11,000x g for 10 min, 10% (wt/vol) polyethylene glycol 8000 (PEG 8000) was added, and the mixture was then incubated at 4°C for 1 h. The supernatant was discarded after centrifugation at 11,000x g for 10 min. Then the pellet was resuspended in 750 μl of SM buffer [100 mM NaCl, 8 mM MgSO4·7H_2_O, 50 mM Tris-Cl (pH 7.5)], and chloroform was added at a ratio of 1:1 (vol/vol), followed by vortexing and then centrifugation at 3,000x g for 15 min. The upper phase was isolated and was added to a polycarbonate centrifuge tube containing 3 ml of 40% glycerol in the lower layer and 4 ml of 5% glycerol in the upper layer. After centrifugation at 151,000x g for 1 h, the supernatant was discarded, and the pellet was resuspended in 400 μl of SM buffer. For animal experiments, any contaminating lipopolysaccharide (LPS) was removed before treatment. Triton X-114 (Sigma, USA) was added to phage solution at a final concentration of 1% (vol/vol) and mixed using vortex for 10 s. After incubation on ice for 10 min, the mixture was transferred to 37°C water bath and incubated for 1 min. After a centrifugation at 15,000x g for 5 min, supernatant was collected and used as the final phage solution. Purified phages were stored at 4°C until use. Standard double agar overlay plaque technique was used for phage enumeration (Kropinski et al., 2009).

### Transmission electron microscopy (TEM) of phage particles

Purified phage sample was loaded onto a copper grid for 1 min followed by negative staining with 2% (vol/vol) uranyl acetate (pH 6.7) and drying. The phage morphology was observed using a Carl Zeiss LIBRA 120 EF-TEM (Carl Zeiss, Oberkochen, Germany) at an accelerating voltage of 120 kV.

### Genome sequencing, annotation, and analysis

Whole genome sequencing of phage DNA was carried out using PacBio RS II system in Macrogen, Korea. Open reading frames (ORFs) were searched using NCBI ORF Finder. Genomic sequence similarity comparison was done using MAUVE. ORF map was drawn using CLC Genomics Workbench 10. Genomic tree was drawn using Mega 7.

### One step multiplication

One step growth experiment was described previously (Hyman and Abedon, 2009). Briefly, phage PBAB08 or PBAB25 was added to a fresh culture of *A. baumannii* at an MOI 0.001 and allowed to adsorb for 5 min. The mixture was then centrifuged at 1,738 xg for 10 min. The supernatant was discarded to remove any free phages and the pellet was resuspended in 3 ml of fresh LB broth incubated at 37°C with shaking. One hundred micro liters of sample was removed from the culture every 5 min and subjected to titration using double-layer agar plate methods (Kropinski et al., 2009). This assay was performed at least in triplicate.

### Phage stability

For temperature stability test, phage titer was measured after incubation of phage lysates for 1 h at different temperatures (4, 37, 45, 55, 60, 65, or 80°C) using a double-layer agar plate method (Kropinski et al., 2009). For pH stability test, phage titer was measured after 1 h's incubation of phage lysates at room temperature by mixing with equal volume of pH buffer solutions of different pH values (pH 3, 4, 5, 6, 7, 8, 9, 10, and 11). pH buffer solutions were made by adding HCl drop by drop to 1 M NaOH solution. The resulting pH was measured using S220 seven compact™ pH/Ion (Mettler Toledo, Columbus, USA).

### Phage protection studies using a mice model

Six week-old female Balb/c mice were obtained from Young Bio, Korea. Mice were divided into 4 groups; Group 1 was treated with SM buffer only. Group 2 was infected with MDR *A. baumannii* (strain 28) only. Group 3 was treated with the phage cocktail only. Group 4 was infected with *A. baumannii* and treated with the phage cocktail. Each group contained 20 mice. Mice were intraperitoneally injected with cyclophosphamide (Sigma, USA) at the concentration of 150 mg/kg at −1 and −3 days of bacterial infection. Before intranasal bacterial or phage injection, mice were anesthetized with 125 mg/kg of avertin (Sigma, USA) by intraperitoneal injection. 1 × 10^8^ CFU of *A. baumannii* was intranasally injected to each mouse at 0 and +1 days. SM buffer was injected to the control group mice instead. 1 × 10^9^ PFU of phage cocktail was intranasally injected to experimental mice every day from −1 to +7 days. At days 0 and +1, phages were injected 4 h after bacterial injection. Survival of mice was observed until 7 days after bacterial infection. Bacterial load in mice lung was counted as follows; lung was isolated at +3 and +4 days and weight was measured. After homogenization, 500 μl of SM buffer was added and was subjected to centrifugation at 2,000x g. The supernatant was used for counting *A. baumannii* in a medium containing both kanamycin (3.5 mg/ml) and ampicillin (5 mg/ml).

### Cytokine, IgE, and histamine assays

To see immune reactions against phages, the cocktail was introduced every day for 7 days to mice in three different routes; intraperitoneal, intranasal, and oral. Three mice were used for each route. Control group was treated with SM buffer. One day after the last treatment of a phage cocktail, mice were sacrificed and serum was obtained for analysis of cytokine profile using Multi-Analyte ELISArray kits (Qiagen, USA), of IgE profile using RayBio^®;^ Mouse IgE ELISA Kit (RayBiotech, USA), and of histamine using Histamine Enzyme Immunoassay Kit (SPI Bio, France).

### Statistical analyses

Student's *t*-test (Bailey, 1959) was used to calculate the significance of the difference among test groups. For each assay, all determinations were carried out at least in triplicate. Statistically significant values were defined as *P* < 0.05 or *P* < 0.01.

## Results

### Antibiotic resistance of clinically isolated *A. baumannii* strains

Fourteen bacterial isolates were selected and their antibiotic resistance and phage susceptibility were observed (Table 1). All the isolates were multidrug-resistant (MDR) strains. Many of them were resistant even to colistin or tigecycline. The 14 *A. baumannii* isolates showed five sequence types (STs) based on MLST (Table 1), and they belonged to the same clonal complex (CC), global complex II. Nine *Acinetobacter* bacteriophages, PBAB05, PBAB06, PBAB07, PBAB08, PBAB25, PBAB68, PBAB80, PBAB87, and PBAB93, were selected from the Bacteriophage Bank of Korea and tested for infection to the clinical *A. baumannii* strains. Seven out of 14 isolates could be infected by four or five phages. The phage susceptibility was not related to antibiotic resistance profile. Isolate 28 was susceptible to all five phages and thus selected for mice infection experiments later. We further characterized two of the phages PBAB08 and PBAB25 from which whole genomic DNA sequences were successfully obtained as single contigs after next generation sequencing.

### Bacteriophage characterization

**Figure 1:**
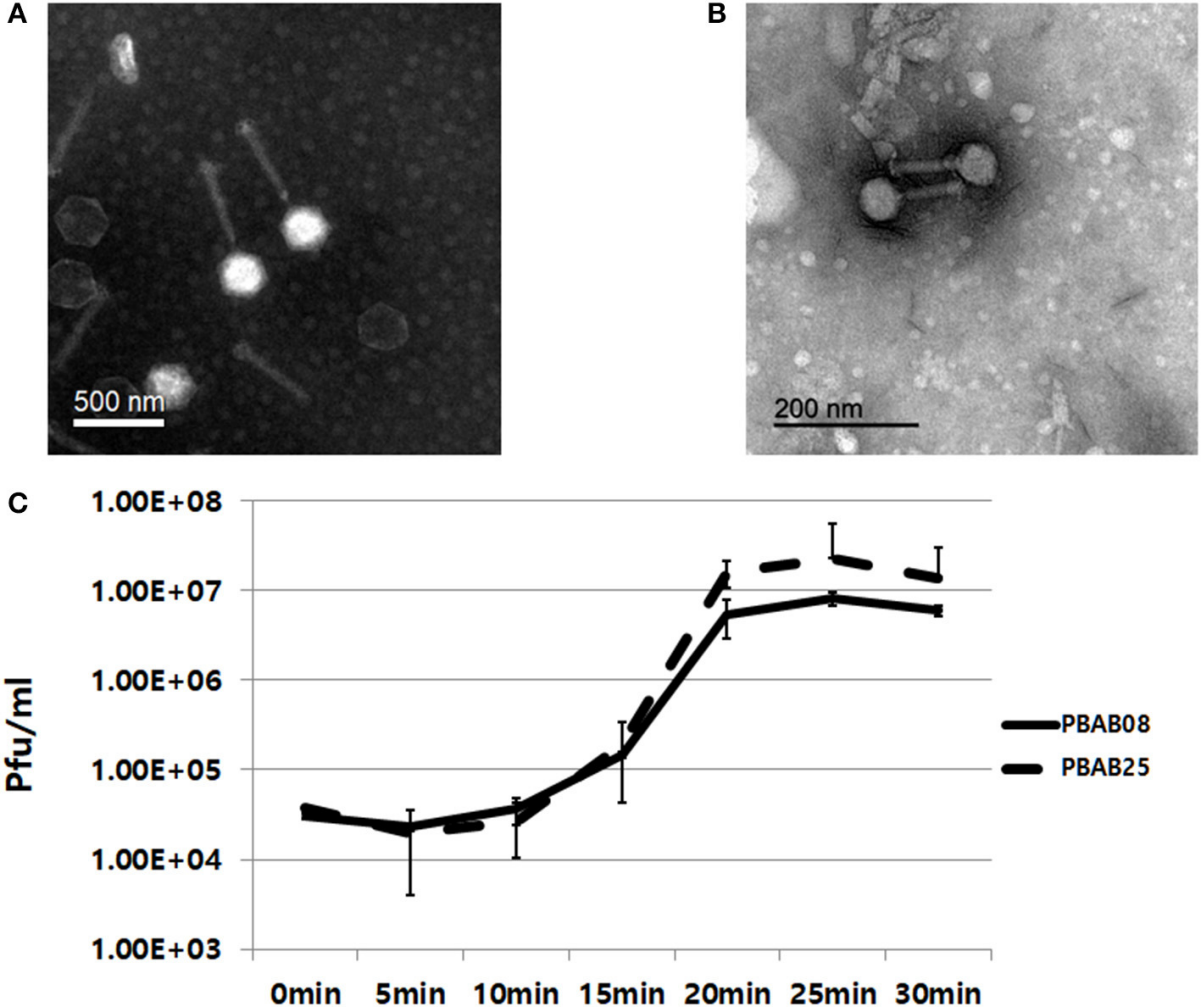
Transmission electron micrographs of bacteriophages PBAB08 **(A)** and PBAB25 **(B)**. Samples were negatively stained with uranyl acetate. Scale bar is shown in each picture. **(C)** One step multiplication of phages PBAB08 (solid line) and PBAB25 (broken line).

Two phages, PBAB08 and PBAB25, belonged to family Myoviridae of order Caudovirales (Figures 1A,B). Their virions were composed of a spherical head and a long, rigid tail. Phage PBAB08 had a head of 180 nm in diameter and a tail of 360 nm in length. The smaller phage PBAB25 had a head of 80 nm in diameter and a tail of 90 nm in length. A complete lysis of host bacteria infected with PBAB08 or PBAB25 occurred in 25 min post infection (Figure 1C). Burst size for each phage was 215 and 630, respectively.

**Figure 2:**
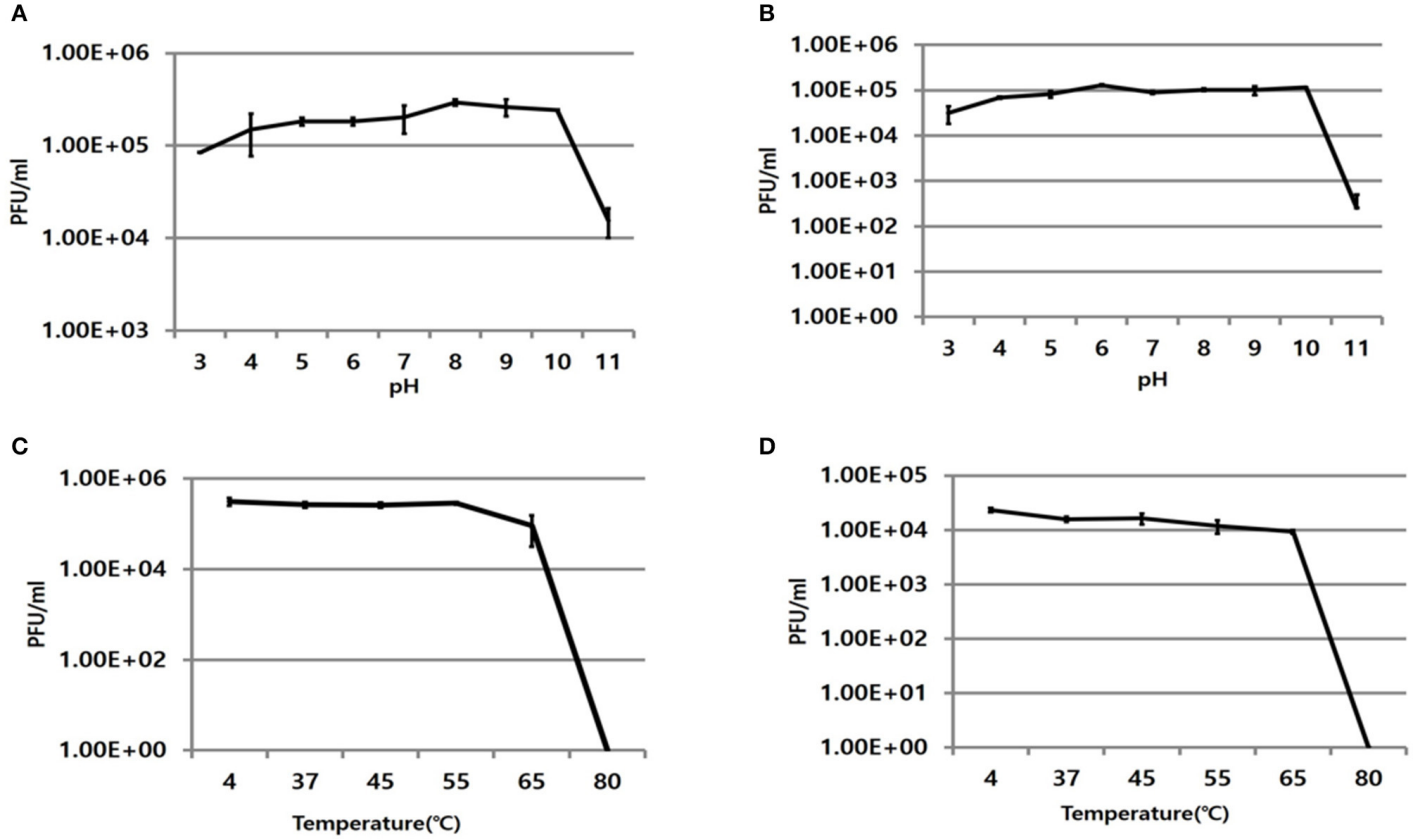
Stability of phages at various pHs and temperatures. Remaining infectivity of phages PBAB08 **(A)** and PBAB25 **(B)** were measured after exposure of phages to indicated pHs for 1 h. Remaining infectivity of phages PBAB08 **(C)** and PBAB25 **(D)** were measured after exposure of phages to indicated temperatures for 1 h. The experiments were carried out in a triplicate.

The infectivity of PBAB08 and PBAB25 remained intact when exposed to pHs ranging from 5 to 10 for 1 h (Figures 2A,B). It decreased rapidly at pHs above 10. The infectivity of both phages remained intact when exposed to temperatures ranging from 4 to 55°C for 1 h and dropped rapidly above 65°C (Figures 2C,D).

### Genomic sequence analysis

**Figure 3:**
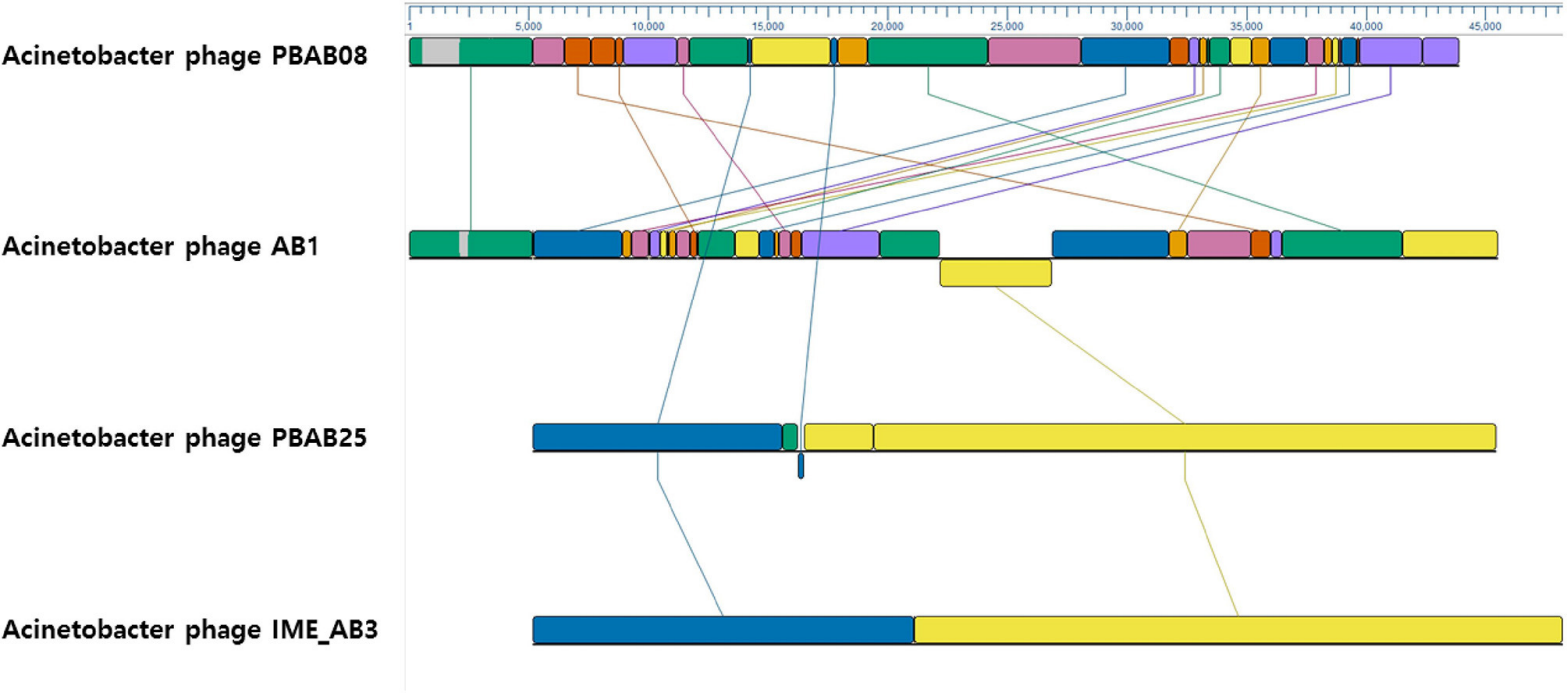
Genomic sequence comparison between phages PBAB08, AB1, PBAB25, and IME_AB3 using MAUVE. Line-connected colored boxes indicate regions of sequence similarity in corresponding phage genomes.

The whole genomes of phages PBAB08 and PBAB25 were sequenced (GenBank accession numbers MG366114 and MG366115, respectively). Their genomes were double stranded linear DNAs of 42,312 base pairs and 40,260 base pairs, respectively. The genome sequences were compared to reported *Acinetobacter* phages using BLASTN. PBAB08 showed a 99% similarity with 57% sequence coverage to phage AB1 (Yang et al., 2010), and PBAB25 showed a 97% similarity with 78% sequence coverage to phage IME_AB3 (Zhang et al., 2015). BLASTN significant alignment coverage of all other known phages were <30%. A mosaic structure between the genomes of PBAB08 and AB1, and that of PBAB25 and IME_AB3 were observed (Figure 3). An extensive rearrangement occurred between genomes of PBAB08 and AB1, while genomic structure was conserved between PBAB25 and IME_AB3.

**Figure 4:**
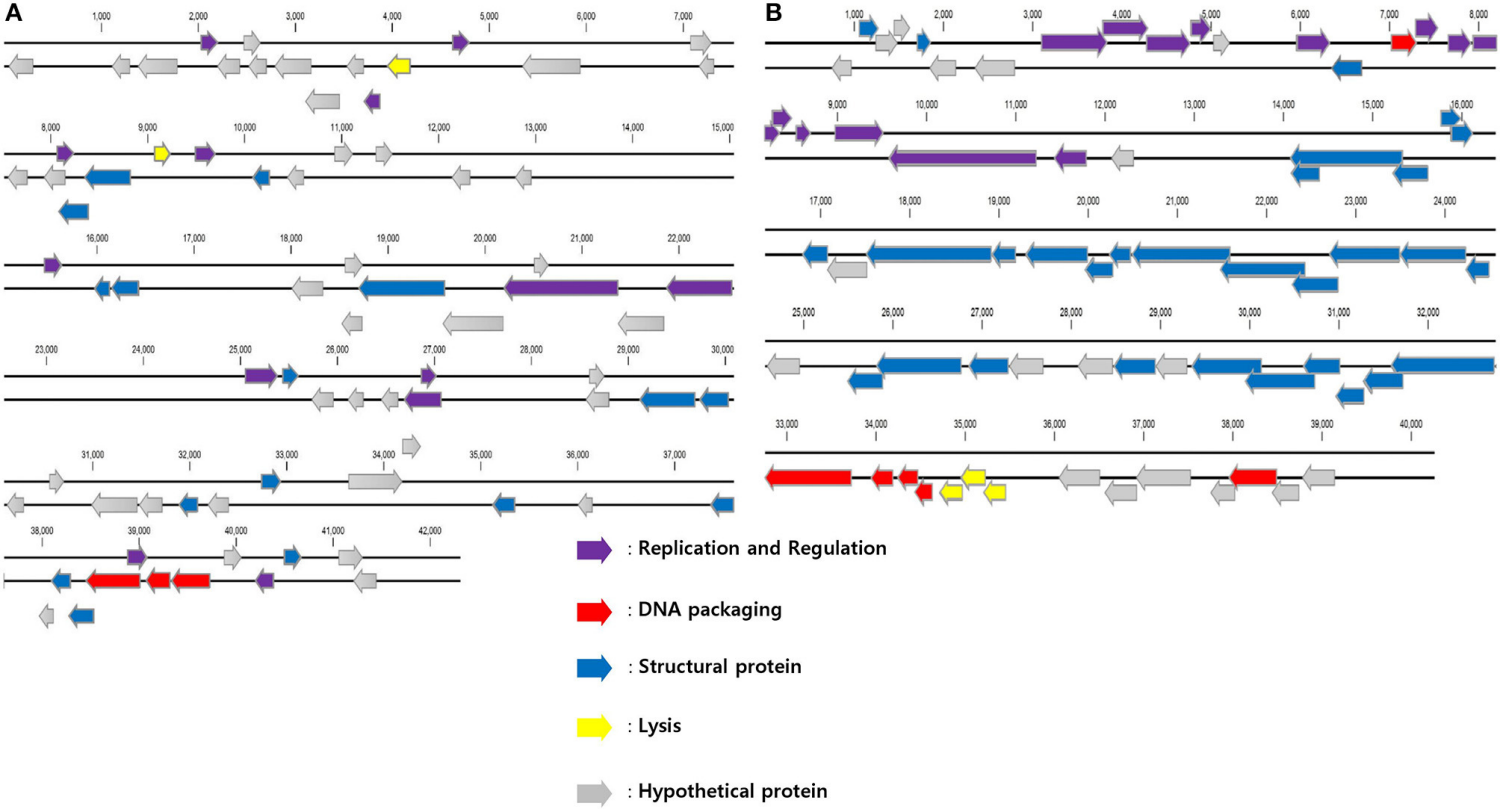
Putative open reading frame (ORF) map of phages PBAB08 **(A)** and PBAB25 **(B)**. ORF map was drawn using CLC Genomics Workbench 10. Each arrow is color-coded according to annotated genomic function.

**Figure 5:**
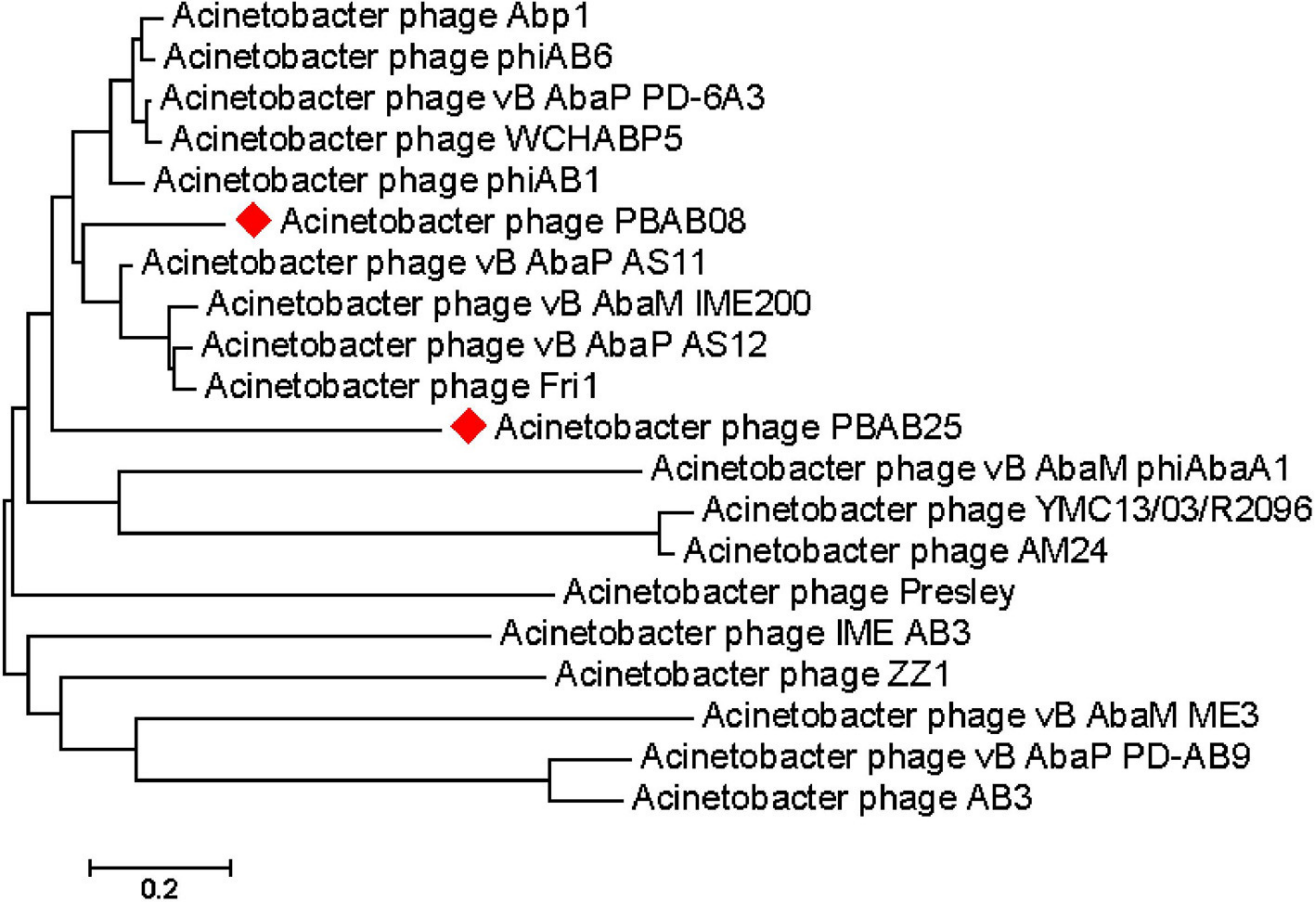
Genomic tree of *Acinetobacter* phages found in GenBank. The tree was drawn based on each phage's DNA polymerase gene sequence using Mega 7.

Seventy six and seventy genes encoding phage proteins were found in the genomes of PBAB08 and PBAB25, respectively (Figure 4). Functional annotation of putative ORFs of each phage is shown in Tables 2, 3. They are divided into four categories according to functions; structural proteins, those involved in DNA packaging, those involved in replication and regulation, and those involved in lysis. It was evident that sequence similarities for both pairs were more prominent in virion proteins than in proteins involved in replication and regulation. It is not fair to compare virion proteins of the two pairs in parallel since the extent of annotations were different. Nevertheless, it is notable that PBAB25 had a wide array of tail proteins suggesting a complicated tail structure. Since both phages had their own putative DNA polymerases, a phylogenetic tree of closely related *Acinetobacter* phages in GenBank was drawn based on the sequence comparison of their DNA polymerase genes (Figure 5). The phages could be grouped as two, in which PBAB08 belonged to one group (upper 10 phages in Figure 5), while PBAB25 belonged to the other group (lower 10 phages). No DNA polymerase was found in functional annotation of ORFs of phage AB1, thus it was not included in the tree.

**Table 2:** Functional annotation of putative ORFs found in bacteriophage PBAB08.

**ORF**	**Function**	**Related bacteria or phage**	**Putative encoded phage protein**	**Similarity (%)**
9	Structural protein	*Acinetobacter* phage WCHABP5	Putative internal virion protein B	100
11		*Acinetobacter* phage vB_AbaP_PD-6A3	Putative internal virion core protein	91
33		*Acinetobacter* phage phiAB6	Putative internal virion protein	75
46		*Acinetobacter* phage AB3	Tail tubular protein B	100
56		*Acinetobacter* phage YMC11/12/R2315	Putative head protein	92
61		*Acinetobacter* phage phiAC-1	Putative capsid protein	95
66		*Acinetobacter* phage phiAC-1	Putative tail fiber protein	88
68		*Acinetobacter* phage AB3	Putative internal virion protein B	73
69		*Acinetobacter* phage AB3	Putative internal virion protein B	97
73		*Acinetobacter* phage IME-AB2	Putative phage head portal protein	93
79		*Acinetobacter* bacteriophage AP22	Putative portal protein	91
86		*Acinetobacter* phage phiAC-1	Putative capsid protein	95
99		*Acinetobacter* phage IME-AB2	Putative phage head portal protein	95
100		*Acinetobacter* phage YMC-13-01-C62	Putative portal protein	98
111		*Acinetobacter* phage YMC-13-01-C62	Putative portal protein	90
114		*Acinetobacter* baumannii 99063	Putative membrane protein	94
121		*Acinetobacter* phage WCHABP12	Putative tail fiber protein	75
54	DNA packaging	*Acinetobacter* phage IME-AB2	Putative phage terminase large subunit	98
78		*Acinetobacter* phage IME-AB2	Putative phage terminase large subunit	93
110		*Acinetobacter* phage IME-AB2	Putative phage terminase large subunit	90
2	Replication and Regulation	*Harpegnathos saltator*	Apolipoprotein D	77
7		*Acinetobacter* phage vB_AbaM_IME200	DNA polymerase I	73
16		*Stenotrophomonas maltophilia*	Adenine phosphoribosyltransferase (fragment)	63
20		*Acinetobacter* phage vB_AbaM_IME200	Carboxypeptidase	95
24		*Massilia* sp. JS1662	Amino acid transporter	50
29		*Staphylococcus aureus* subsp. *aureus*	Nickel ABC transporter, nickel/metallophore periplasmic binding domain protein	89
40		*Rathayibacter* sp. VKM Ac-2630	Transcriptional regulator	75
52		*Burkholderia* sp. WSM4176	TonB-dependent receptor	60
109		*Acinetobacter* phage WCHABP12	Global DNA-binding transcriptional dual regulator	87
116		*Acinetobacter* phage YMC-13-01-C62	Putative RNA polymerase	92
119		*Acinetobacter* phage YMC-13-01-C62	Putative baseplate assembly protein	98
120		*Acinetobacter* phage WCHABP1	Baseplate J-like protein	98
125		*Acinetobacter* phage WCHABP1	Putative RNA polymerase	89
19	Lysis	*Acinetobacter* phage WCHABP5	Putative holin	51
74		*Acinetobacter* phage WCHABP5	Putative holin	83

**Table 3:** Functional annotation of putative ORFs found in PBAB25.

**ORF**	**Function**	**Relative bacteria or phage**	**Putative encoded phage protein**	**Similarity**
46	Structural protein	*Acinetobacter* phage vB_AbaM_ME3	Baseplate hub protein	97
67		*Acinetobacter* phage vB_AbaM_ME3	Head completion protein	100
77		*Acinetobacter* phage vB_AbaM_ME3	Baseplate hub protein	100
106		*Acinetobacter* phage IME_AB3	Putative portal protein	100
107		*Acinetobacter* phage IME_AB3	Putative scaffold protein	91
109		unclassified Siphoviridae	Putative tail terminator protein	72
110		*Acinetobacter* phage IME_AB3	Putative major tail tube protein	98
112		*Acinetobacter* phage IME_AB3	Putative tail chaperonin protein	100
113		*Acinetobacter* phage IME_AB3	Putative tail tape measure protein	87
114		unclassified Siphoviridae	Putative distal tail protein	86
116		*Acinetobacter* phage IME_AB3	Putative capsid and scaffold protein	100
135		*Acinetobacter* phage IME_AB3	Putative head protein	99
136		*Acinetobacter* phage IME_AB3	Putative major capsid protein	100
138		*Acinetobacter* phage IME_AB3	Putative structural protein	95
141		*Acinetobacter* phage IME_AB3	Putative tail tape measure protein	94
142		unclassified Siphoviridae	Putative distal tail protein	86
144		*Acinetobacter* phage IME_AB3	Putative tail protein	91
167		*Acinetobacter* phage IME_AB3	Putative portal protein	100
168		*Acinetobacter* phage IME_AB3	Putative head protein	98
171		*Acinetobacter* phage IME_AB3	Putative major tail tube protein	98
172		*Acinetobacter* phage IME_AB3	Putative tail completion protein	100
173		*Acinetobacter* phage IME_AB3	Putative tail tape measure protein	67
174		*Acinetobacter* phage IME_AB3	Putative tail tape measure protein	100
175		*Acinetobacter* phage IME_AB3	Putative capsid and scaffold protein	99
176		*Acinetobacter* phage IME_AB3	Putative capsid and scaffold protein	100
177		*Acinetobacter* phage IME_AB3	Putative tail protein	100
178		*Acinetobacter* phage IME_AB3	Putative tail protein	99
179		*Acinetobacter* phage IME_AB3	Putative tail protein	90
184		*Acinetobacter* phage vB_AbaM_ME3	Putative membrane protein	98
38	DNA packaging	*Acinetobacter* phage IME_AB3	Putative RecB exonuclease	100
101		*Acinetobacter* phage IME_AB3	Putative terminase small subunit	97
103		*Acinetobacter* phage IME_AB3	Putative terminase large subunit	96
128		*Acinetobacter* phage IME_AB3	Putative endonuclease	81
133		*Acinetobacter* phage IME_AB3	Putative terminase large subunit	97
166		*Acinetobacter* phage IME_AB3	Putative terminase small subunit	94
5	Replication and regulation	*Acinetobacter* phage IME_AB3	Putative replicative primase/helicase	97
9		*Acinetobacter* phage IME_AB3	Putative single-stranded DNA-binding protein	100
10		*Acinetobacter* phage IME_AB3	Putative DNA/RNA helicase protein	84
11		*Acinetobacter* phage IME_AB3	Putative DNA polymerase subunit	95
36		*Acinetobacter* phage IME_AB3	Putative replicative primase/helicase	99
37		*Acinetobacter* phage IME_AB3	Putative MazG pyrophosphatase	88
39		*Acinetobacter* phage IME_AB3	Putative DNA/RNA helicase protein	42
40		*Acinetobacter* phage IME_AB3	Putative DNA polymerase subunit	99
71		*Acinetobacter* phage IME_AB3	Putative replicative primase/helicase	100
73		*Acinetobacter* phage vB_AbaM_ME3	Putative DNA polymerase subunit	100
74		*Acinetobacter* phage IME_AB3	Putative DNA polymerase subunit	84
121		*Acinetobacter* phage vB_AbaM_ME3	Adenine/guanine phosphoribosyltransferase	97
147		*Acinetobacter* phage vB_AbaM_ME3	Chemical-damaging agent resistance protein C	94
131	Lysis	unclassified Siphoviridae	Putative holin, class II	78
132		*Acinetobacter* phage IME_AB3	Putative endolysin	99
165		*Acinetobacter* phage IME_AB3	putative endolysin	99

### *In vivo* efficacy of phage cocktail

**Figure 6:**
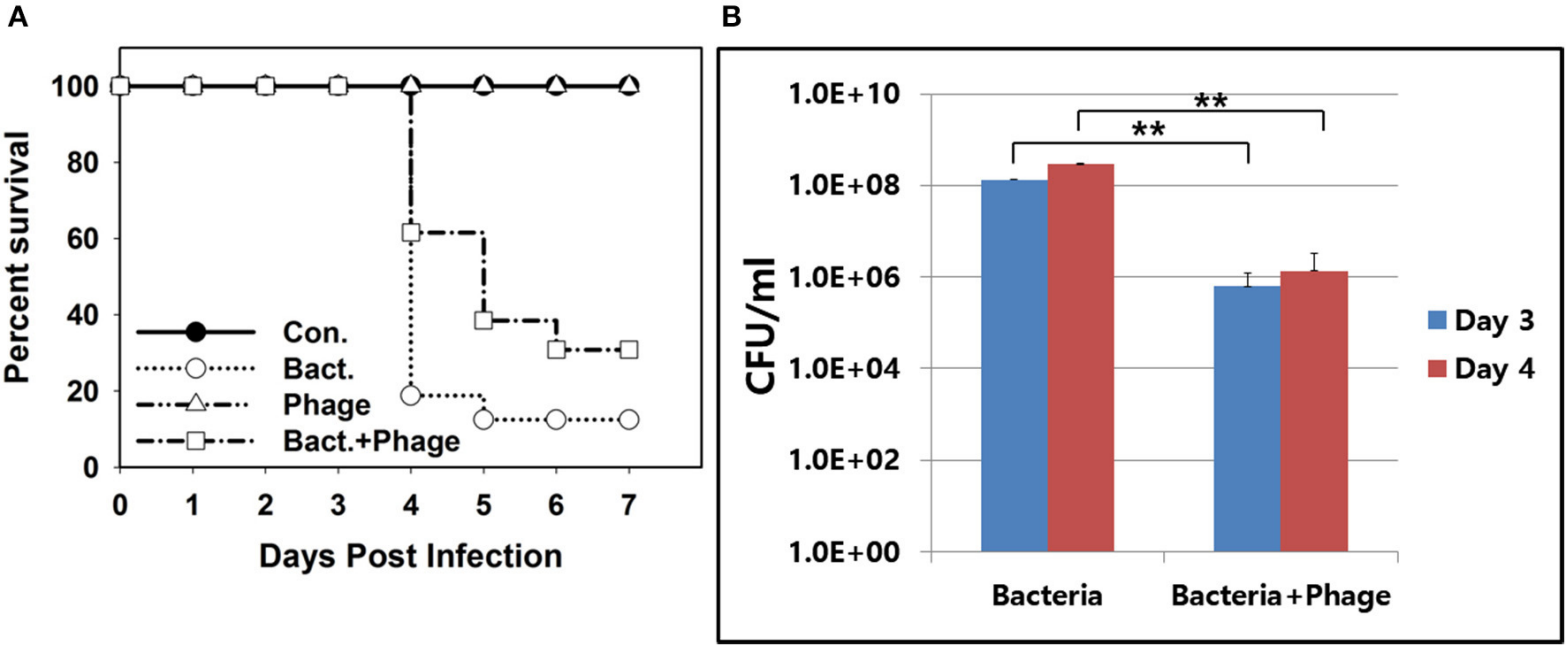
*In vivo* efficacy of the phage cocktail containing PBAB08 and PBAB25 in a mice nasal infection model. **(A)** Survival of 4 groups of mice observed for 7 days after bacterial infection. Group 1 (closed circle) was inoculated with SM buffer only. Group 2 (open circle) was infected with MDR *A. baumannii* strain 28 only. Group 3 (triangle) was treated with the phage cocktail only. Group 4 (rectangle) was infected with *A. baumannii* and treated with the phage cocktail. **(B)** Bacterial load in lungs of infected mice in 3 and 4 days post-infection with or without phage treatment. ^**^P < 0.01.

Only 15% of mice infected with *A. baumannii* intranasally survived at 7 days post infection (Figure 6A). On the contrary, 35% of mice infected with the bacteria followed by treatment with the phage cocktail survived. A better survival of phage-treated mice was observed at day 4 post infection. Sixty percentage of mice survived with phage treatment while 20% of mice survived with only bacterial infection. Mice treated with phage only remained healthy for the entire experimental period.

Reduction of bacterial load in infected lung of mice was observed (Figure 6B). At 3 and 4 days post bacterial infection, bacteria resistant to both kanamycin and ampicillin were counted. Double-resistant bacterial count was reduced more than 100-fold for both days. Nevertheless, it was clear that a complete elimination of *A. baumannii* was not achieved with this phage treatment.

### Immune reactions against the phage cocktail

**Figure 7:**
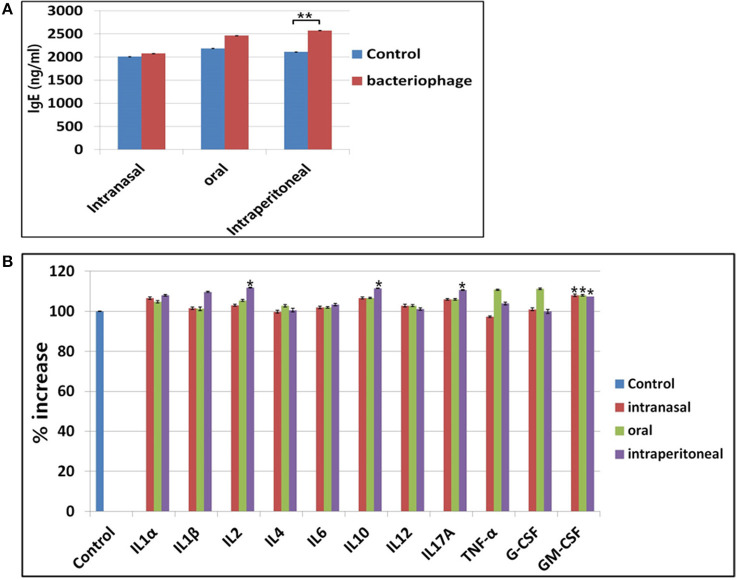
Observation of immune responses against phage treatment in mice. **(A)** Changes in IgE production after treatment of phages in three different routes. **(B)** Changes in cytokine production after phage treatment in intranasal, oral, or intraperitoneal route. ^*^P < 0.05, ^**^P < 0.01.

Phages are foreign substances to mice and there are chances they could elicit immune responses. We checked changes in serum IgE level indicating any allergic responses for three different routes of phage injection (Figure 7A). A 20% increase in serum IgE was observed for intraperitoneal injection route, while no significant change was observed for either intranasal or oral administration. For cytokines, a slight increase of serum GM-CSF was observed for all three routes of phage administration (Figure 7B). In intraperitoneal route, slight increases of IL2, IL10, and IL17A were also observed. Thus, inflammatory response against the phage cocktail was minimal. No significant change in histamine level was observed when phage cocktail was administered (Supplementary Figure 1).

## Discussion

Among the clinically isolated MDR *A. baumannii* strains used in this study, some of them were infected by three or more phages, while the others were not infected by any of the five phages used. The degree of drug-resistance of a host bacterium was not related to the number of phages infecting the same host, indicating that acquirement of drug resistance, probably by horizontal gene transfer (Huang et al., 2015; Krahn et al., 2016; Zhang et al., 2017) did not involve alteration of phage receptor or replication mechanisms. It further suggested that MDR bacteria could be treated with phages as long as infecting phages could be isolated.

Head size of PBAB08 (180 nm) was twice that of PBAB25 (80 nm). Yet, the lengths of genomic DNAs of the two phages were about the same. For example, the genome size of phage T4 is 169 kilobase pair and the head size varies between 85 and 120 nm (Keller et al., 1988). Although a minimal requirement should be enough room for containing entire phage genomic DNA, it seems that there are other determinants of the head size of a phage. In the case of T4, head size determinant included scaffolding core and the shell of the procapsid, mutants of which resulted in varied head sizes (Keller et al., 1988).

From pH and temperature stability data, phage infectivity should remain intact at temperatures and pHs reaching well outside normal human physiological conditions. This should be a good characteristics if the phages are intended for therapeutic applications. A phage preparation in a suitable buffer kept in a refrigerator could remain stable until it encounters target bacteria when applied *in vivo*.

A mosaic in genomic structure observed from closely related phages in this study is consistent with the modular theory of phage evolution where phage genomes are mosaics of modules that recombines freely in genetic exchanges involving different phages (Botstein, 1980). It was also observed in other viruses including P2-like phages (Davies and Lee, 2006) and even animal viruses (Botstein, 1980). It is no wonder that viruses evolve rapidly by exchanging genetic materials and extend their diversity. The ease of such exchange in a host cell could be one reason why phages are the most divergent biological entities on earth. In the case of phage AB1 which lacks its own DNA polymerase gene, the phage should depend on host DNA polymerase for its own replication. Still its genome retains genomic mosaic to PBAB08, suggesting that modular recombination among phage genomes was independent of their replication strategy. It should be noted that DNA polymerases of PBAB25 and phage Presley was closest in sequence, but overall similarity between their genomic sequences was <30%. It suggests that multiple rounds of recombination events has occurred among various strains of phages during the evolution.

It is noteworthy that only 3 genes encoding putative tail proteins in PBAB08 with a longer tail was annotated while 14 genes annotated in PBAB25 with much shorter tail. It seems that tail length is not related to complexity of its structure. Although electron microscopy of phage IME_AB3, closest to PBAB25 in genomic DNA sequence, was not clear enough to tell the tail structure (Zhang et al., 2015), the two closely related phages of IME_AB3, *Acinetobacter* phage vB_AbaS_Loki (Turner et al., 2017) and *Achromobacter* phage phiAxp-1 (Li et al., 2016), have noncontractile tails and are classified as Siphoviridae, while PBAB25 belonged to Myoviridae. Thus it suggests that sequence similarity of tail components does not ensure the similarity in tail morphology.

More than two-fold increase of survival from mice treated with phage cocktail was shown in this study. Although it proved a limited effectiveness, there could be a room for improvements. In a previous report, mice survival after phage treatment in a nasal infection model was higher than this experiment (Jeon et al., 2016b). But it should be noted that in the same report mice were inoculated with phages only after 30 min of bacterial infection, in which bacterial proliferation time was much shorter before confronting phages. In this study, there was a 4 h gap between bacterial infection and phage inoculation. In another report, open wound in rats were treated with phages after *A. baumannii* infection (Kusradze et al., 2016). Phage treatment was done at 12 h after bacterial infection, but showed a higher reduction of bacterial load than this study. Depending on types of infection and routes of inoculation, the outcome seems to vary. A pretreatment of phages in this study did not help efficiently eliminating incoming bacteria. Most of the phages could be destabilized and lost their infectivity before bacterial infection. In a wound infection model, loss of phages during application would be less than nasal infection model since there would be less chance of immune-mediated clearance. Also, the amount of phages applied seems to be critical. A dose dependency was observed in a previous report (Jeon et al., 2016b).

Increase in serum proinflammaotry cytokines after phage treatment, if any, could reflect a possible inflammatory response elicited by phages. But it was minimal in this study. Mice remained healthy and behaved normally. The observation is coherent with the previous finding that oral inoculation of T7 phages induced a minimal increase in cytokine production of mice (Park et al., 2014). Another phage, SH-Ab 15519, was reported and the therapeutic efficacy was better than the phage cocktail used in this study (Hua et al., 2018). Application of only a single phage showed 90% survival of mice with the M.O.I of 1 or 10. BLAST search revealed 24% coverage with 92% identity when compared to PBAB08, meaning only a low degree of relatedness. As in this study, application of SG-Ab 15519 was generally safe based on histopathological examination of mice lung treated with the phage and cytokine analysis.

Taken together, bacteriophage treatment for mice nasally infected with clinically isolated MDR *A. baumannii* strain was proven effective and safe. Depending on bacterial strains and phages, an optimal condition for an effective treatment needs to be set. Parameters should include selection of phages from a bank collection based on susceptibility of the causative bacterial strain, composition of a cocktail containing different phage-resistant group members, doses and lengths of phage applications, and routes of phage application. A rapid inoculation of phages after exposure to the pathogen is another critical requirement.

Based on findings that the phage cocktail containing PBAB08 and PBAB25 is effective and safe for treating infections by MDR *A. baumannii in vivo*, phage therapy would be a viable alternative for antibiotics, especially in cases where antibiotics are not treatment options any more.

## Author contributions

HM and KK: designed experiments; KC, YJ, WK, and GH: carried out experiments; HO and JJ: analyzed experimental results; HM: wrote the manuscript.

### Conflict of interest statement

The authors declare that the research was conducted in the absence of any commercial or financial relationships that could be construed as a potential conflict of interest.

## Supplementary Material

Supplementary Figure 1 Histamine levels in sera from mice treated with phages using three different routes, intraperitoneal (i.p), intranasal (i.n), or oral (p.o). The experiment was performed in triplicate. **P* < 0.05, ***P* < 0.01.
