# Corrigendum: Characterization of Two Novel Bacteriophages Infecting Multidrug-Resistant (MDR) *Acinetobacter baumannii* and Evaluation of Their Therapeutic Efficacy *in vivo*

**DOI:** 10.3389/fmicb.2021.815173

**Published:** 2022-01-05

**Authors:** Kyoungeun Cha, Hynu K. Oh, Jae Y. Jang, Yunyeol Jo, Won K. Kim, Geon U. Ha, Kwan S. Ko, Heejoon Myung

**Affiliations:** ^1^Department of Bioscience and Biotechnology, Hankuk University of Foreign Studies, Yong-In, South Korea; ^2^The Bacteriophage Bank of Korea, Hankuk University of Foreign Studies, Yong-In, South Korea; ^3^Samsung Medical Center, Sungkyukwan University School of Medicine, Suwon, South Korea

**Keywords:** multidrug-resistance, *Acinetobacter baumannii*, bacteriophage therapy, mouse model, genome analysis

In the original article, there was an error. We mistakenly misspelled histamine as “histamin” in a heading in the Materials and Methods section. A correction has been made to **Materials and Methods**, where the heading **Cytokine, IgE, and Histamine Assays** now appears correctly.

In the original article, there was an error. We mistakenly omitted the statement “No significant change in histamine level was observed when phage cocktail was administered (Supplementary Figure 1).” in the Results section.

**Supplementary Figure 1:**
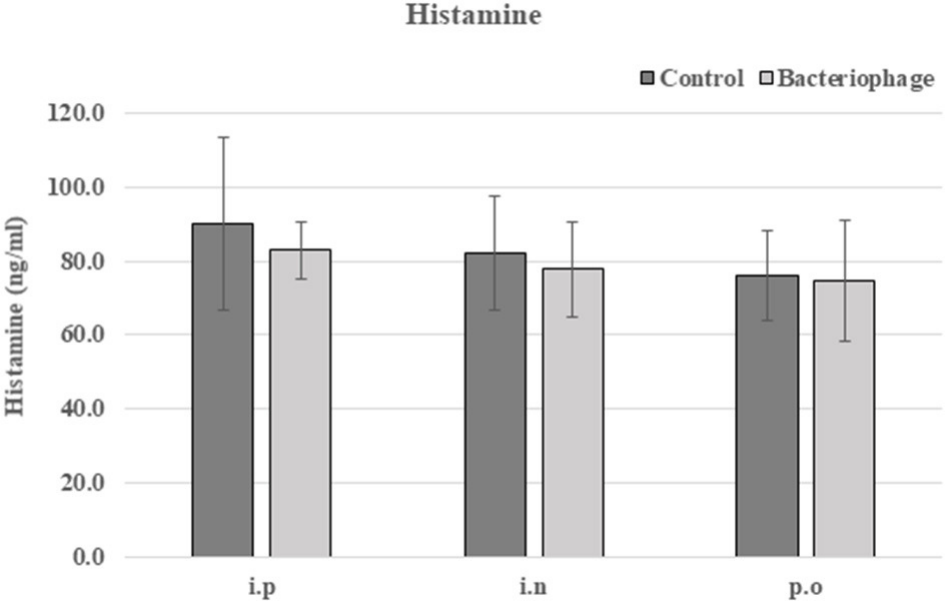
Histamine levels in sera from mice treated with phages using three different routes, intraperitoneal (i.p), intranasal (i.n), or oral (p.o). The experiment was performed in triplicate. *P < 0.05, **P < 0.01.

A correction has been made to **Results, Immune Reactions Against the Phage Cocktail**, paragraph 1:

“Phages are foreign substances to mice and there are chances they could elicit immune responses. We checked changes in serum IgE level indicating any allergic responses for three different routes of phage injection (Figure 7A). A 20% increase in serum IgE was observed for intraperitoneal injection route, while no significant change was observed for either intranasal or oral administration. For cytokines, a slight increase of serum GM-CSF was observed for all three routes of phage administration (Figure 7B). In intraperitoneal route, slight increases of IL2, IL10, and IL17A were also observed. Thus, inflammatory response against the phage cocktail was minimal. No significant change in histamine level was observed when phage cocktail was administered (Supplementary Figure 1).”

Accordingly, the added Supplementary Figure 1 appears below.

The authors apologize for these errors and state that this does not change the scientific conclusions of the article in any way. The original article has been updated and Supplementary Figure 1 is now included.

## Publisher's Note

All claims expressed in this article are solely those of the authors and do not necessarily represent those of their affiliated organizations, or those of the publisher, the editors and the reviewers. Any product that may be evaluated in this article, or claim that may be made by its manufacturer, is not guaranteed or endorsed by the publisher.

