# Supplementary figures and images for: Characterization of Two Novel Bacteriophages Infecting Multidrug-Resistant (MDR) Acinetobacter baumannii and Evaluation of Their Therapeutic Efficacy in Vivo
Source: Front Microbiol. 2018 Apr 10;9:696. doi: 10.3389/fmicb.2018.00696 (PMC5932359; doi:10.3389/fmicb.2018.00696)

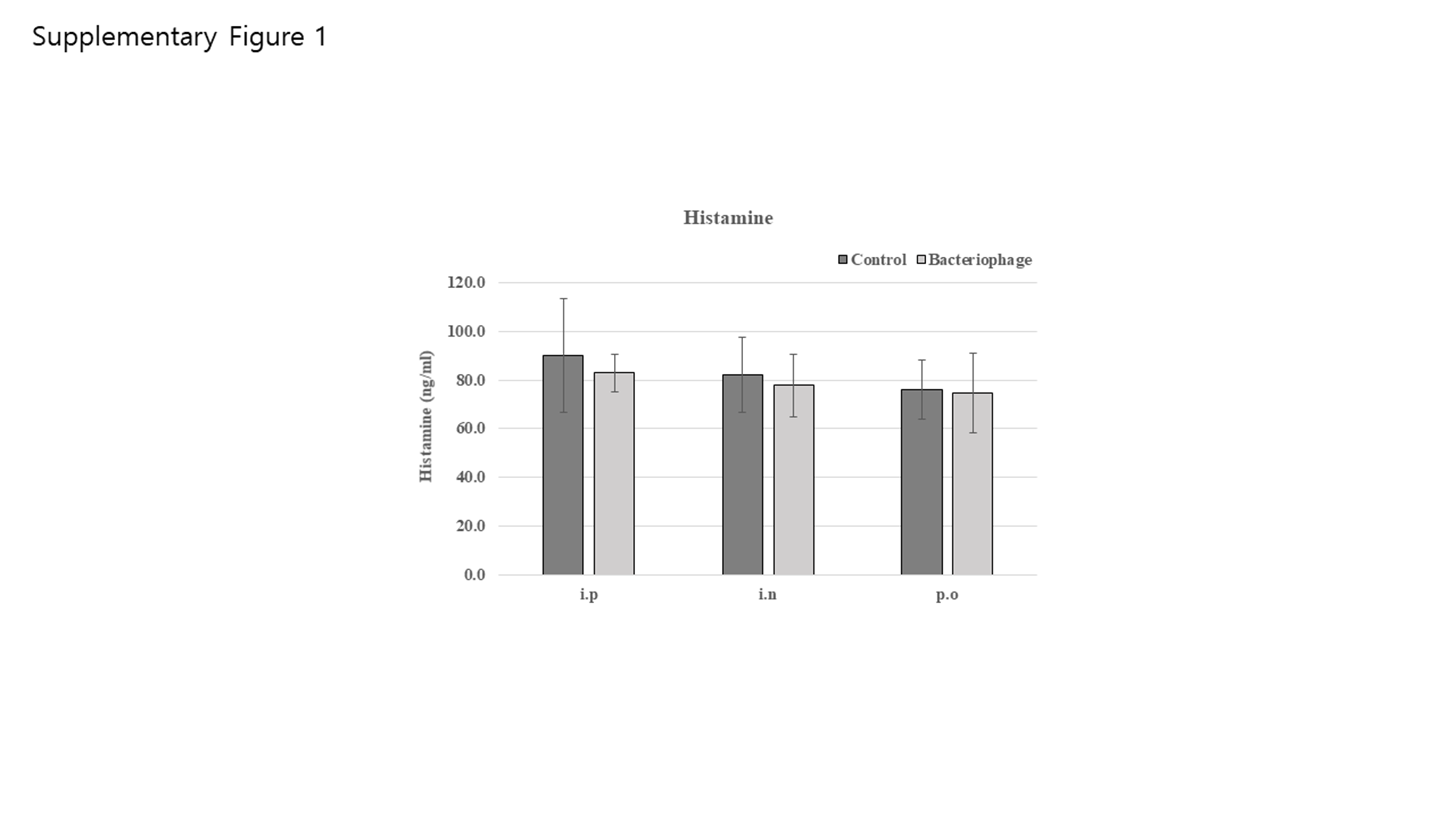

Supplement: Supplementary Figure 1 — Histamine levels in sera from mice treated with phages using three different routes, intraperitoneal (i.p), intranasal (i.n), or oral (p.o). The experiment was performed in triplicate. *P < 0.05, **P < 0.01. [file Image_1.TIF]
